# Cultural openness and desire to learn in relation to ethnocultural empathy among university students in multilingual contexts

**DOI:** 10.3389/fpsyg.2025.1463349

**Published:** 2025-06-18

**Authors:** Beatriz Peña-Acuña, Carmen M. Toscano-Fuentes, Patricia Flor-Arasil

**Affiliations:** ^1^Department of Philology, Faculty of Education, Universidad de Huelva, Huelva, Spain; ^2^Department of English Philology, Faculty of Education, Universidad de Huelva, Huelva, Spain; ^3^Faculty of Health Sciences, Universidad Internacional de Valencia, Valencia, Spain

**Keywords:** intercultural education, learning, migrants, multiculturalism, students, socialization, teaching, social behavior

## Abstract

**Introduction:**

Cultural openness and the desire to learn have been examined through the lens of inclusive education, particularly in relation to multicultural competencies and empathy. Empathy is recognized as a key factor in enhancing group relations. However, there is a lack of research on how these variables interact among university students in multilingual contexts.

**Methods:**

This quantitative study surveyed 530 Spanish university students using a validated questionnaire based on the Everyday Multicultural Competencies/Revised Scale of Ethnocultural Empathy. The statistical analyses employed included Spearman’s correlation coefficient, the Mann-Whitney *U* test, and linear regression.

**Results:**

Results revealed a strong positive correlation between cultural openness, desire to learn, and empathy. Statistically significant gender differences were also found in levels of empathy and openness.

**Discussion:**

These findings suggest that training adult students in cultural openness may foster greater empathy and support inclusive education. Implementing activities that promote ethnocultural empathy in university classrooms could inform curriculum development for multicultural teacher training programs.

## 1 Introduction

Due to ongoing migration flows, university classrooms have experienced a significant rise in migrant and international student enrollment, fostering environments rich in cultural diversity (Athanasopoulou et al., 2018; Kustati et al., 2020) and varied communicative contexts (Munezane, 2021; Van Werven et al., 2021). This multiculturalism has promoted societal changes toward pluralism (Peterson, 2020).

Despite a growing body of literature on multicultural education, there is still a lack of empirical research that explores how variables such as intercultural sensitivity, cultural openness, and ethnocultural empathy operate in multilingual university classrooms, particularly in the Spanish context. This study addresses this gap by examining how these dimensions influence student experiences and teaching practices in culturally diverse academic environments.

In this research, we focus on two closely related constructs: cultural openness and ethnocultural empathy. Cultural openness is defined as the willingness to acknowledge and appreciate cultural differences and similarities, and to engage with them, thereby fostering positive intergroup relations. On the other hand, ethnocultural empathy involves understanding and emotionally connecting with people from different cultural backgrounds. Although they are conceptually distinct, we hypothesize that greater cultural openness promotes the development of ethnocultural empathy by fostering inclusive attitudes and emotional responsiveness toward cultural diversity.

### 1.1 Intercultural competence

In this context, fostering intercultural relationships is crucial because they strengthen bonds among participants, facilitate integration, and preserve cultural identity (Martínez-Otero Pérez, 2001; Szelei et al., 2020; Toledo Jofré, 2012; United Nations Educational, Scientific and Cultural Organization, 2008). However, discrimination and prejudice, often stemming from a lack of empathy and cultural openness, can challenge these relationships. Thus, linguistic competence alone is insufficient. Participants must also develop intercultural sensitivity, which Boudouaia et al. (2022, p. 3) explain as “emotions and feelings towards cultural differences involving tolerance and respect,” and intercultural communicative competence which includes “recognizing one’s cultural identities, understanding others, maintaining a positive attitude, and interacting effectively” (Banjongjit and Boonmoh, 2018, p. 79).

Professors play a central role in promoting these competencies by creating safe, inclusive environments (Gu, 2016; Pino Castillo et al., 2020; United Nations Educational, Scientific and Cultural Organization, 2019) and fostering multicultural knowledge (Stanton, 2015). Through pedagogies that respect diverse backgrounds (Ladson-Billings, 1995) and the promotion of democratic values (del Gil Pino et al., 2017; Terhart and von Dewitz, 2018), they can cultivate empathy, positive attitudes, and respect (Kustati et al., 2020; Yilmaz, 2016). Nevertheless, communication barriers (Tapia Parada and Tour, 2022), limited professional training (Choi and Mao, 2021), and professors’ own beliefs and cultural perceptions (Hanna, 2020, 2022; McAllister and Irvine, 2023; Mendenhall et al., 2017; Rodríguez-Izquierdo et al., 2020) can hinder these efforts. Hence, specific training is needed to strengthen teachers’ abilities to meet multilingual and multicultural needs (Toscano Fuentes and Legaz-Torregrosa, 2022; Ryan et al., 2019).

Thus, pre-service teacher education should prioritize two objectives: training in intercultural competence to foster cultural sensitivity and effective interaction (Rojas-Barreto, 2019; Cuartas Álvarez, 2020; Esteban-Núñez, 2020; Gay and Kirkland, 2013; Toscano Fuentes and Legaz-Torregrosa, 2022), and preparing teachers to “adapt to societal transitions [becoming] proactive agents of social change” (Tuomi, 2005, p. 207). Educators also learn from “the experience of having immigrant classmates, seen as culturally enriching” (Níkleva and Ortega-Martín, 2015, p. 315), fostering tolerance, critical thinking, and peaceful environments (Firdaus et al., 2020) while reducing miscommunication across cultures (Chavez, 2020; Song, 2019; Tabatadze and Gorgadze, 2018). Teachers’ beliefs can evolve through experience with migrants (Haim and Tannenbaum, 2022; Rodríguez-Izquierdo et al., 2020; Silverman, 2010).

### 1.2 Intercultural sensitivity

Educational institutions are becoming increasingly diverse (Van Werven et al., 2021), making the development of intercultural competence essential. Students’ intercultural sensitivity—involving cultural awareness, willingness to learn, and positive attitudes (Bhawuk and Brislin, 2000; Boudouaia et al., 2022)—is seen as an emotional dimension (Taylor, 1994) and a prerequisite for intercultural competence (Hammer et al., 2003; Mostafaei Alaei and Nosrati, 2018; Sarwari and Abdul, 2017). However, it is important to recognize that intercultural sensitivity can have both positive and negative effects, as cultural psychology classes may foster either cultural sensitivity or cultural stereotyping (Buchtel, 2014).

Intercultural sensitivity in language education is essential for developing communicative competencies, attitudes, and values that facilitate understanding, dialogue, and effective interaction among people from different cultures (Byram and Wagner, 2018). However, there are ongoing debates (Luo, 2024) about the most suitable approaches, models, and methods (Vaca, 2022) to implement it in educational practice.

### 1.3 Cultural openness

According to Nesdale and Todd (2000) cultural openness is identified as the willingness to be open and interested in the differences and similarities between one’s own group and others, reducing ethnocentrism and promoting positive intergroup attitudes. Qaisi (2021) described it as a practice of inclusion. Based on Finck et al.’s (2021) study, Peña-Acuña (2024, p. 1) explained that cultural openness is a sub-dimension within multicultural competencies, part of a range of cultural attitudes and skills (Escalante Rivera et al., 2014), including vision, affections, and beliefs. This sub-dimension, along with cultural references (Jiménez Vargas et al., 2017) and cultural knowledge and behaviors (Álvarez Baz, 2013), forms the basis for understanding multicultural competencies.

Nonetheless, individuals with cultural openness must also demonstrate empathy towards the situation and other group members.

The relevance of cultural openness in educational contexts is underscored by Chen and Starosta (2000), who noted its enhancement of self-esteem, self-monitoring, open-mindedness, empathy, interaction involvement, and non-judgmental attitudes, which can facilitate effective classroom management practices (Boudouaia et al., 2022). According to Yuen and Grossman (2009), individuals must encounter cultural differences to develop intercultural sensitivity, necessitating teachers to first recognize their own worldviews before assisting students in cultivating intercultural understanding. Peña-Acuña and Cislowska (2024) demonstrated the importance of fostering cultural openness among pre-service teachers to enhance multicultural competence and empathy. They outlined benefits such as heightened awareness of classroom diversity, development of intercultural skills to collaborate effectively with families, preparation for interpersonal challenges in educational settings, and integration of multicultural content.

### 1.4 Ethnocultural empathy

Meneses and Larkin (2012) adopt Stein (1917) original concept of empathy. They define it as the direct understanding of another person’s experience and recognition of its ‘otherness’, distinguishing it from current models of empathic comprehension which categorize it as either ‘intellectual’ or ‘sympathetic’ experiences. Similarly, Nesdale et al. (2005, p. 624) develop the concept of empathy as “the ability to experience the same feelings as those of another person in response to a particular situation.” Ginsberg et al. (2018, p. 251) argue that “understanding the experiences of migrant learners enhances empathy, enabling educators to effectively support them through a ‘pedagogy of recognition.” McAllister and Irvine (2023) assert that educators’ empathy facilitates more positive interactions, supportive classroom environments, and student-centered practices.

Therefore, ethnocultural empathy, as explained in the study by Finck et al. (2021), can be seen as a significant mediator in fostering positive intergroup attitudes through intergroup contact (Pettigrew et al., 2011), promoting cultural openness. Shunchao and Thangadurai (2023) stated that when students interact with individuals from various backgrounds, ethnocultural empathy improves, fostering social cohesion and a deeper understanding of each other’s cultural heritage. Kapıkıran (2021) indicates that ethnocultural empathy is best predicted by universality-diversity orientation, low immigrant discrimination, empathy-prosocial behavior, threat perception towards outgroups, and ethnicity-religion politics. For the sake of brevity, this study will henceforth refer to this term simply as empathy, as the variable.

In summary, although prior research has examined cultural openness and empathy individually, the interaction between these two constructs, particularly within multilingual university settings, remains poorly understood. Exploring the connection between cultural openness and empathy is essential for promoting inclusive educational environments. Therefore, the present study focuses on analyzing their relationship through the following objectives:

- To determine the association between the variables cultural openness and desire to learn and the empathy variable.
- To ascertain if there are sex differences concerning the variables cultural openness and desire to learn.
- To investigate whether cultural openness and desire to learn can be predicted by considering empathy and sex.

The hypotheses proposed are as follows:

First Hypothesis

Null Hypothesis (H_0_): There is no correlation between cultural openness and empathy.

Alternative Hypothesis (H_1_): There is a correlation between cultural openness and empathy.

Second Hypothesis

Null Hypothesis (H_0_): There are no sex differences among participants regarding cultural openness.

Alternative Hypothesis (H_2_): There are sex differences among participants regarding cultural openness.

Third Hypothesis

Null Hypothesis (H_0_): Cultural openness cannot be predicted considering the variables of empathy and sex.

Alternative Hypothesis (H_3_): Cultural openness can be predicted considering the variables of empathy and sex.

## 2 Methodology

This is an exploratory quantitative study. The primary goal of exploratory research is to investigate phenomena, identify patterns, generate hypotheses, and gain an initial understanding of the relationships between variables. The advantage of this methodology lies in its objectivity, positivism, reliability, and validity (León and Montero, 2020, p. 449).

### 2.1 Participants

The sample included 530 Spanish university students belonging to various universities (University of Huelva, University of Alicante, University of Almería, University of Cáceres, University of Castilla La Mancha, University of Granada, University of Las Palmas de Gran Canaria, University of Los Lagos, University of Málaga, University of Oviedo, University of Sevilla, University of Valladolid, University of Zaragoza, University of Madrid, University Rey Juan Carlos). The types of studies they pursue include humanities, philology, social sciences or educational studies. They are in a position to learn a foreign language. Erasmus Plus program funds and promotes the learning of a foreign language. The participants were selected through a convenience sampling strategy, as the questionnaire was distributed via an online link to accessible and willing students.

From the total of 530 Spanish university students in this sample, women represented a higher proportion (75.7%) compared to men (24.3%). Although the sample provides valuable insights into the studied phenomena, this notable gender imbalance may limit the generalizability of the findings, particularly regarding sex-based comparisons. Future studies should aim for a more balanced gender representation to strengthen the external validity of the results (Table 1).

**Table 1:** Sex of participants.

		Frequency	Percentage	Valid percentage	Cumulative percentage
Valid	Female	401	75.7	75.7	75.7
	Male	129	24.3	24.3	100.0
	Total	530	100.0	100.0	

The participants’ ages ranged from 18 to 55 years, with a mean age of 22.53 years and a standard deviation of 3.83, indicating a moderate dispersion.

### 2.2 Tools and materials

The participants performed voluntarily by completing the Finck et al. (2021) questionnaire, administered through Google Forms and hosted via a link on the Moodle educational management platform. The data collected were exported to Excel and then processed using SPSS v.25 (IBM).

The Multicultural Competencies and Empathy Inventory, originally validated by Finck et al. (2021) in Colombia, was adapted to Spanish for this study. It consists of 47 items rated on a Likert scale from 1 (strongly disagree) to 6 (strongly agree). Although the full instrument is organized into five dimensions, only the items related to two constructs — cultural openness and desire to learn (hereinafter referred to as cultural openness) and empathy — were analyzed.

Although the original instrument was validated by Finck et al. (2021), preliminary analyses were conducted to assess its reliability and factorial structure within the Spanish university student population. The internal consistency was satisfactory (Cronbach’s alpha > 0.80 for all scales), and an exploratory factor analysis supported the expected structure. These results provide initial evidence of the cross-cultural applicability of the instrument in the present context.

### 2.3 Procedure of data analysis

Different statistical tests have been used to study the quantitative data in depth. In the first phase, descriptive tests have been used. According to Pérez Juste et al. (2012, p. 4) these tests are informative and “highlight a representative characteristic of the group.” Next, three inferential tests have been used in combination. Using these tests, according to Pérez Juste et al. (2012), one obtains “a series of conclusions about some aspect or variable present in a population from observations of behaviors in one or more samples” (p. 6).

The choice of statistical tests was made after examining the normality of the data. First, the nonparametric Spearman correlation test is applied. Spearman’s correlation measures the strength and direction of the monotonic relationship between two variables, whether it is negative or positive. It does not assume a linear relationship between the variables. Second, the nonparametric U-Mann–Whitney test for independent samples. This test compares the distributions of two independent groups to determine if there are significant differences in their distributions. It does not assume normality or equal variances. Third, linear regression is included to model and predict the relationship between variables, identifying the strength and direction of the relationship. It can also be used to infer potential causal influences between variables, assuming certain conditions met.

Regarding missing data, an initial screening was conducted prior to the statistical analyses. The dataset did not exhibit any substantial missing values. In the few instances where individual responses were missing, listwise deletion was employed, meaning that incomplete cases were excluded from the specific analyses in which the missing data occurred. This method was implemented to maintain the internal consistency and integrity of the regression models.

Additionally, to assess the potential influence of outliers, Cook’s distance values were examined. All values were below the commonly accepted threshold of 1, indicating that no individual data points exerted undue influence on the estimated model parameters. Durbin-Watson statistics ranged from 0.000 to 0.038, suggesting no evidence of autocorrelation or residual patterns that would violate the assumptions underlying the regression analysis.

## 3 Results

Descriptive statistics about the participants (Sex, Age) were performed. Also, according to the Kolmogorov–Smirnov test in relation to the cultural openness and empathy variables, it was found that the data did not follow a normal distribution.

Considering a non-normal distribution, the direct correlation inferential test with Spearman’s Rho was performed to evaluate monotonic relationships between cultural openness and empathy. The results of the correlational analysis are shown in Table 2. The existing relationship is direct and very strong, therefore, the linear regression test is appropriate to be carried out.

**Table 2:** Spearman’s test.

			Factor score cultural openness	Factor score empathy
Spearman’s rho	Factor Score Cultural Openness	Correlation coefficient	1	1.000^**^
		Sig. (two-tailed)		0
		*N*	530	530
	Factor Score Empathy	Correlation coefficient	1.000^**^	1
		Sig. (two-tailed)	0	
		*N*	530	530


** The correlation is significant at the 0.01 level (two-tailed).

The Whitney U-Mann test is conducted for independent samples with respect to the sex variable and the cultural openness variable. This test is performed in relation to sex with respect to the cultural openness variable considering two groups (female sex and male sex). Results are shown in Table 3, where statistical significance differences appear (*p*-value < 0.05) rejecting the null hypothesis assuming that there are differences between both groups and they are significant.

**Table 3:** *U*-Mann Whitney (Cultural Openness variable).

Statistic	Factor score Cultural Openness
*U-*Mann–Whitney	22.430
Z	–2.27
Asymptotic significance (two-tailed)	0.023
Grouping variable	Sex

Additionally, while the statistical tests indicate significant differences, the Cohen’s d value for the gender differences in cultural openness suggests a moderate effect size (d = 0.5), indicating that the difference between male and female participants, while statistically significant, represents a moderate practical difference in cultural openness. This highlights that, although the observed effects are significant, they may not be large enough to have profound real-world implications. Nevertheless, they are noteworthy for understanding gender-based cultural openness.

Likewise, the results of the U-Mann Whitney test indicate that there are significant differences in the measured variable between the groups. The median for male gender was −0.331, whilst the median for female gender was 0.116. Specifically, they suggest that there is a significant difference in cultural openness between males and females. The median for females was significantly higher (0.116) compared to the median for males (−0.331), indicating that, in general, females tend to have greater cultural openness compared to males. This significant difference in medians suggests that women may be more willing or have a greater propensity to experiment with and adopt new ideas, perspectives, or cultures compared to men. It is represented in Table 4.

**Table 4:** *U*-Mann Whitney (sex variable).

	Sex	
	Female	Male
	Mean	Mean
Factor Score Cultural Openness	0.11612	–0.33125

Linear regression tests are also performed between the variables cultural openness, empathy and sex looking for the predictive character between them. Cultural openness (independent) and empathy (dependent) are tested in both groups together with the gender variable. The following regression equation is proposed:

Cultural Openness=b0+a(Empathy)+Sex

Observing the correlation matrix from Table 5 we can observe that there exists correlation between sex and both cultural openness and empathy, including this relation to be statistically significant.

**Table 5:** Correlations between cultural openness, empathy and sex.

Correlations					
			Factor score cultural openness	Factor score empathy	Sex
Spearman’s rho	Factor score cultural openness	Correlation coefficient	1.000	1.000*	–0.099*
		Sig. (two-tailed)		0.000	0.023
		*N*	530	530	530
	Factor score empathy	Correlation coefficient	1.000**	1.000	–0.100*
		Sig. (two-tailed)	0.000		0.022
		*N*	530	530	530
	Sex	Correlation coefficient	–0.099*	–0.100*	1.000
		Sig. (two-tailed)	0.023	0.022	
		*N*	530	530	530

** The correlation is significant at the 0.01 level (two-tailed). * The correlation is significant at the 0.05 level (two-tailed).

As reported by the coefficients from the regression model tested we can propose a linear regression equation model. The coefficients are found in Table 6.

**Table 6:** Coefficients from regression test.

Coefficients^a^											
Model		Unstandardized coefficients		Standardized coefficients	*t*	Sig.	Correlations			Collinearity statistics	
		B	Std. error	Beta			Zero-order	Partial	Part	Tolerance	VIF
1	Constant	-0.011	0.004		−3.011	0.003					
	Factor Score Empathy	1.017	0.000	1.000	2192.639	0.000	1.000	1.000	0.997	0.995	1.005
	Sex	0.006	0.003	0.001	2.011	0.045	−0.073	0.087	0.001	0.995	1.005
Dependent variable: Factor score cultural openness											

According to the equations, from the multiple linear regression analysis presented below, differentiated by sex, men have a higher baseline cultural openness than women, which could be influenced by the scores obtained in the empathy subscale.

- Cultural Openness = (intercept) + female/male + empathy.
- Cultural Openness (female = 1) = −0,011 + 0,006(female) + 1,017(empathy) = − 0,011 + 0,006(1) + 1,017(empathy) = − 0,005 + 1,017 (direct score for the empathy subscale).
- Cultural Openness (male = 2) = − 0,011 + 0,006(male) + 1,017(empathy) = − 0,011 + 0,006(2) + 1,017(empathy) = − 0,011 + 0,012 + 1,017(empathy) = 0,001 + 1,017 (direct score for the empathy subscale).

Regarding the coefficients in the model, sex exhibited a small but statistically significant effect on the dependent variable (*B* = 0.006; *β* = 0.001; t = 2.011; *p* = 0.045). In contrast, the empathy factor demonstrated a strong positive association with cultural openness (*B* = 1.017; β = 1.000; t = 2192.639; *p* < 0.001), suggesting it is a key predictor in the model.

**Figure 1:**
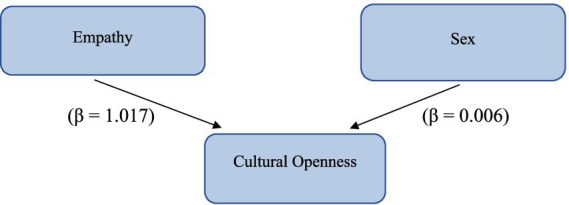
Predictive relationship between empathy, cultural openness and sex.

Figure 1 illustrates the predictive relationship between Empathy and Cultural openness, emphasizing the slightly higher baseline level of cultural openness observed in male participants, as indicated by the regression model. Specifically, for every one-point increase on the empathy scale, cultural openness increases by approximately 1.017 points. This suggests that individuals with higher levels of empathy also tend to exhibit greater cultural openness.

Additionally, the model indicates that both gender and empathy predict cultural openness, but empathy has a significantly larger effect (1.017) than sex (0.006).

Prior to interpreting the regression results, assumptions of linearity, independence of errors, homoscedasticity, and multicollinearity were evaluated. Residual plots suggested no major violations of homoscedasticity, and tolerance values were all above 0.20, indicating no concerns with multicollinearity. Tolerance values range from 0.785 to 0.982, indicating low collinearity. Values below 0.10 indicate severe collinearity, which is not observed in this case.

The VIF (Variance Inflation Factor) values are between 1.018 and 1.274, well below the critical threshold of 10 (and even the more conservative criterion of 5). This suggests that there is no significant presence of collinearity between the variables in the model.

**Figure 2:**
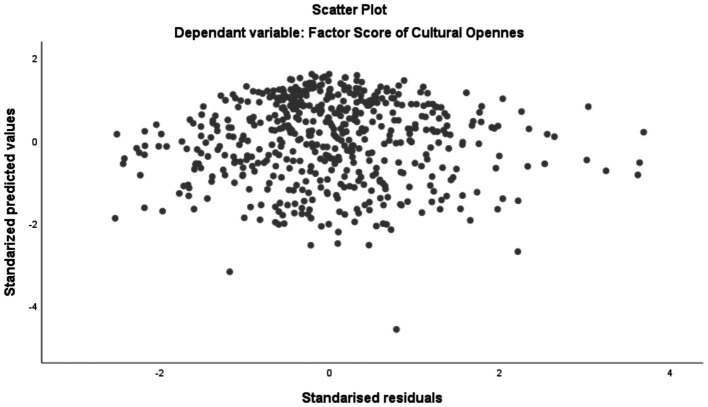
Studentized residuals vs. standardized predictions plot.

To check the homoscedasticity of the residuals, a scatter plot between the standardized residuals and the standardized predicted values was visually inspected. The distribution of the points was random and approximately constant along the horizontal axis, with no evidence of systematic patterns and no evidence of increasing or decreasing variance. Therefore, the homoscedasticity assumption is considered to be met. This issue is depicted in Figure 2.

**Figure 3:**
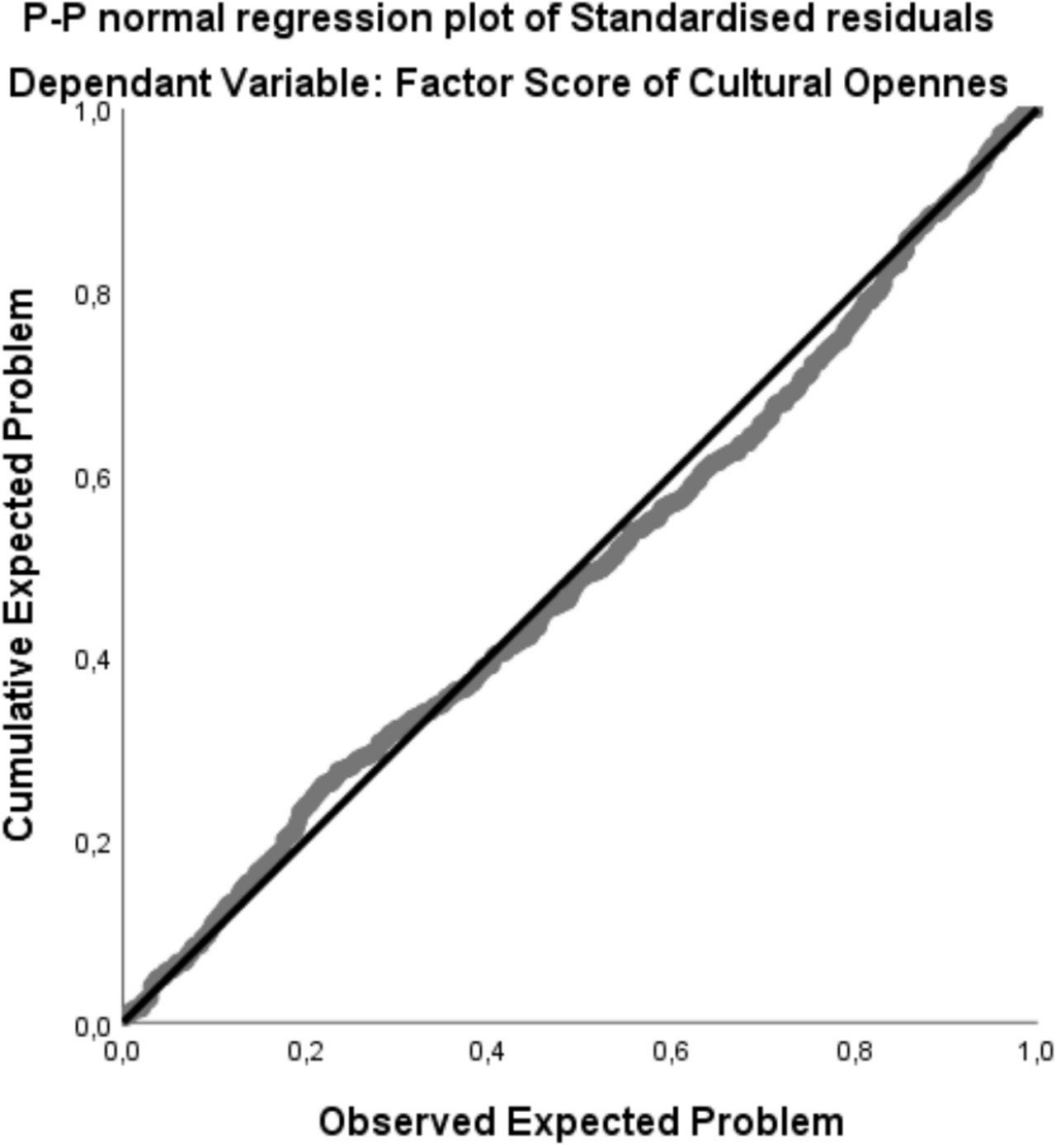
Cumulative expected problem vs. observed expected problem plot.

Furthermore, the distribution of standardized residuals did not show severe deviations from normality, supporting the appropriateness of the linear regression analysis. It is shown in Figure 3.

## 4 Discussion

The alternative hypothesis H_1_ is supported, indicating a strong direct relationship between cultural openness and empathy variables among Spanish university students. This first finding advances the field beyond previous validated questionnaires conducted in the English-speaking context (Mallickrodt et al., 2014) and in the Spanish-speaking context in Colombia (Finck et al., 2021).

Similarly, Peña-Acuña (2024), following a systematic review of cultural openness, argues that these two variables, cultural openness and empathy, are related in the educational context. Peña-Acuña and Cislowska (2024) suggest that, among future teachers, training in cultural openness is crucial for the development of multicultural competencies and empathy.

Similarly, the variable cultural openness (Peña-Acuña, 2024) in language teaching is also closely linked to the intercultural approach and the development of cultural competencies. Yakunina et al. (2012), in their statistical study, argue that the open-mindedness, flexibility, and empathy of international university students in the United States indirectly enhance their acceptance of diversity and adaptation. In this way, Escalante Rivera et al. (2014) mention that cultural openness encompasses a range of cultural attitudes and skills, including vision, affections, and beliefs. Likewise, Dreamson et al. (2017) describe cultural openness in education as a practice of inclusion and the integration of existing educational agents, such as students and teachers. Jiménez et al. (2017) address the integration of cultural references with cultural knowledge and behaviors to fully understand multicultural competencies. McAllister and Irvine (2023), based on a qualitative study, indicate that empathy improves teaching with diverse students, creating a positive and student-centered environment.

The alternative hypothesis H_2_ is confirmed, as there are sex differences among participants regarding cultural openness in Spanish students. Moreover, these differences indicate higher levels of cultural openness in female than male university students, when comparing sex differences focusing exclusively in cultural openness and desire to learn. This is the second major contribution of this study in this field.

The alternative hypothesis H_3_ is confirmed, predicting cultural openness based on the variables of empathy and sex in Spanish university students. Regarding the variable of sex, men exhibit a higher baseline level of cultural openness compared to women, which will be similarly influenced by empathy levels for both genders. It is interesting to mention how empathy levels can influence the cultural openness of female and male, thus when forecasting it seems that the male’s baseline is higher than female’s baseline, in contrast to the findings for the previous hypothesis H_2_. These two assertions constitute the third finding of this research.

Park et al. (2016), in a randomized controlled experiment, assert that empathy reduces selfishness and facilitates a greater appreciation of others’ humanity, more so in a sample of Japanese than Australians, although this effect varies by culture.

Zhao et al. (2021) reveal the importance of considering cultural and gender variables when investigating empathy. Their study explored how culture and sex influence empathy, both as a trait and as a state. They found that this effect varies depending on the characteristics of the stimulus and among different groups of participants, such as Australian women, Australian men, Chinese women, and Chinese men. Regarding gender, the results of a pretest-posttest experimental study by Fernández-Corbacho et al. (2024), which compared the variable of empathy with another variable, anxiety, following an educational intervention with Spanish university students, showed that women exhibited higher levels of empathy than men.

Building on these findings, practical applications could be developed within teacher education programs. Specifically, integrating structured interventions aimed at enhancing empathy and cultural openness could be highly beneficial. For example, curricula could incorporate experiential workshops, role-playing exercises, and intercultural exchange activities that directly foster empathy skills and openness to diverse perspectives. These interventions should be embedded early in the teacher education process to prepare future educators for multicultural classrooms, ultimately promoting inclusivity and improving student outcomes across diverse educational settings.

## 5 Conclusion

In this study of Spanish university students, a direct and significant relationship was found between cultural openness and empathy. Higher levels of cultural openness are associated with greater empathy. This finding fulfills the first objective and goes beyond previous research in both Anglophone and Spanish-speaking contexts. Cultural openness, which involves exploring and understanding other cultures, is linked to empathy, which includes understanding and sharing the emotions of others. Training in cultural openness particularly benefits future teachers by developing multicultural competencies and fostering empathy in the classroom. Additionally, cultural openness is related to language teaching and the intercultural approach. International students with open-mindedness and flexibility show greater acceptance of diversity and better adaptation in diverse cultural environments. In summary, these skills are crucial in higher education and contribute to a positive, student-centered environment.

This study confirms that there are sex differences in cultural openness among Spanish university students. This result addresses the second objective. Men exhibit greater cultural openness compared to women. Furthermore, it is predicted that empathy influences this relationship, affecting both genders equally. This finding fulfills the third objective and is relevant for understanding how culture and sex impact empathy.

Based on the previously discussed literature review, the authors agree that the development and application of cultural openness and empathy are two essential factors in multicultural educational contexts, as they provide a theoretical and practical foundation for fostering inclusion in the classroom.

Despite the valuable insights provided by this study, certain limitations should be considered when interpreting the findings. The cross-sectional design prevents causal inferences, and the predominance of female participants (75.7%) introduces a sampling bias that may limit the generalizability of the results. Furthermore, the contextual specificity of the Spanish university population may restrict the applicability of the findings to broader or more diverse educational contexts. Future research should address these limitations by incorporating longitudinal designs and more balanced, multicultural samples.

### 5.1 Practical application

Based on this study, cultural openness and empathy should be promoted in university teaching contexts. Empathy is essential for student well-being and the building of positive relationships. Teachers can foster empathy by encouraging students to understand others’ perspectives and show empathy toward their peers. In the classroom, activities such as debates, group projects, and discussions can cultivate empathy by allowing students to put themselves in others’ shoes.

Additionally, a focus on cultural diversity in the classroom should be emphasized and valued. Cultural diversity is a reality in university classrooms that can lead to mutual enrichment. Teachers should consider cultural differences and promote intercultural understanding. Students should learn to appreciate and respect different ways of thinking and living.

Promoting tolerance and acceptance is also advisable. Empathy is related to tolerance. By understanding others’ experiences and emotions, students are more likely to accept diversity. Teachers can create an inclusive environment by recognizing and valuing individual differences.

Moreover, to operationalize these findings in educational practice, teacher education programs could integrate specific training modules focused on empathy-building and intercultural competence. For example, role-playing exercises, cultural immersion experiences, and structured reflection activities could be incorporated into curricula to enhance students’ ability to understand diverse perspectives. Workshops aimed at developing intercultural sensitivity and emotional intelligence could also be systematically implemented as part of pre-service teacher training, fostering more inclusive and empathetic classroom environments.

### 5.2 Prospective

Considering the results of the multiple regression analysis in the results section, it is advisable to conduct a mediation/moderation analysis as a future approach to assess the effect between both scales. These analyses should be conducted separately for males and females since they cannot be included together in a mediation/moderation analysis due to their categorical nature.

Additionally, future studies could employ mixed-method approaches with larger samples to further investigate the predictive differences observed between males and females regarding cultural openness and empathy. This approach would isolate other factors, such as cultural influences, and other variables affecting this phenomenon.

### 5.3 Limitations

The limitation of this study lies in not having conducted a pre-test and post-test experimental study with this group, which will be pursued prospectively. Additionally, conducting a comparative exploratory study with samples from both European and non-European countries would have enriched the cultural scope of the findings. Furthermore, combining quantitative and qualitative approaches could have provided deeper insights into the phenomenon and expanded the scope of variables considered.

Given the cross-sectional design of this study, causal relationships cannot be established, and longitudinal changes over time could not be examined. Additionally, as the sample consisted solely of Spanish university students, no cross-cultural comparisons were conducted. Future research should consider employing longitudinal designs and incorporating samples from diverse cultural contexts to strengthen the generalizability and depth of the findings.

## Data Availability

The raw data supporting the conclusions of this article will be made available by the authors, without undue reservation.
